# Supplementary material for: Foliar Fertilization by the Sol-Gel Particles Containing Cu and Zn
Source: Nanomaterials (Basel). 2022 Dec 30;13(1):165. doi: 10.3390/nano13010165 (PMC9824736; doi:10.3390/nano13010165)
Supplement: Supplementary file 1 [file nanomaterials-13-00165-s001.zip › nanomaterials-2120573-supplementary.pdf]

Foliar Fertilization by the Sol-Gel Particles Containing Cu and Zn

Beata Borak [1,*], Krzysztof Gediga [2], Urszula Piszcz [2] and Elżbieta Sacała [2]

[1] Department of Mechanics, Materials and Biomedical Engineering, Faculty of Mechanical Engineering, Wroclaw University of Science and Technology, Smoluchowskiego Str. 25, 50-370 Wroclaw, Poland
[2] Department of Plant Nutrition, Institute of Soil Science, Plant Nutrition and Environmental Protection, The Faculty of Life Sciences and Technology, Grunwaldzka Str. 53, 50-357 Wroclaw, Poland
* Correspondence: beata.borak@pwr.edu.pl

Figure S1. DLS measurements

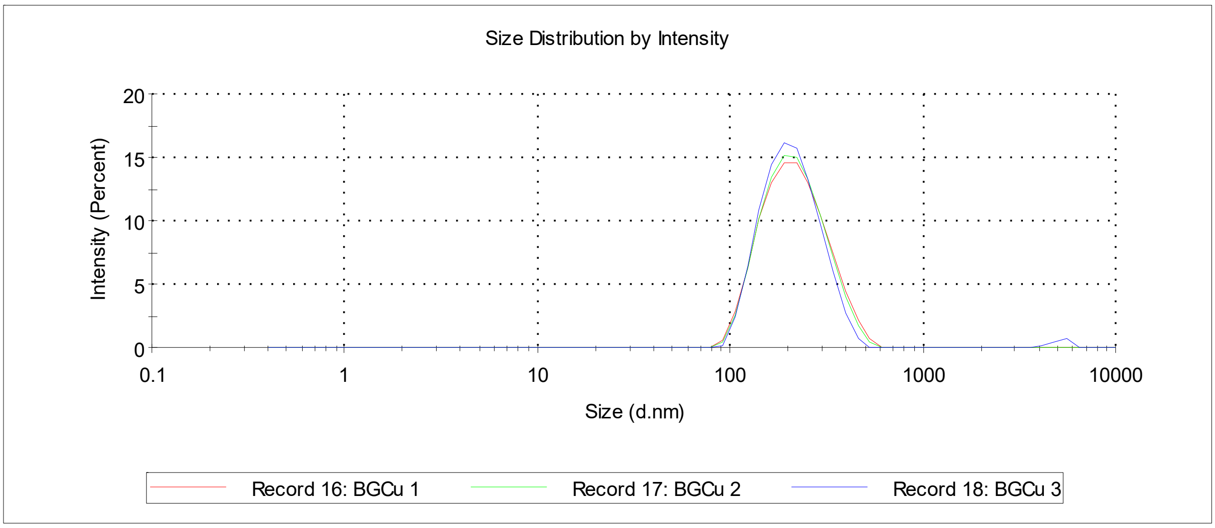

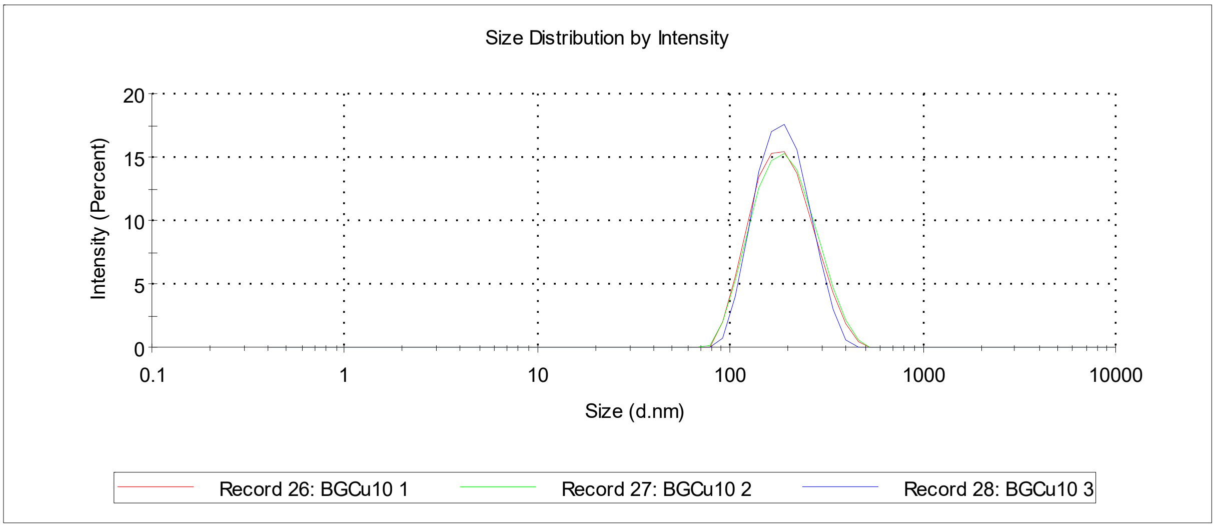

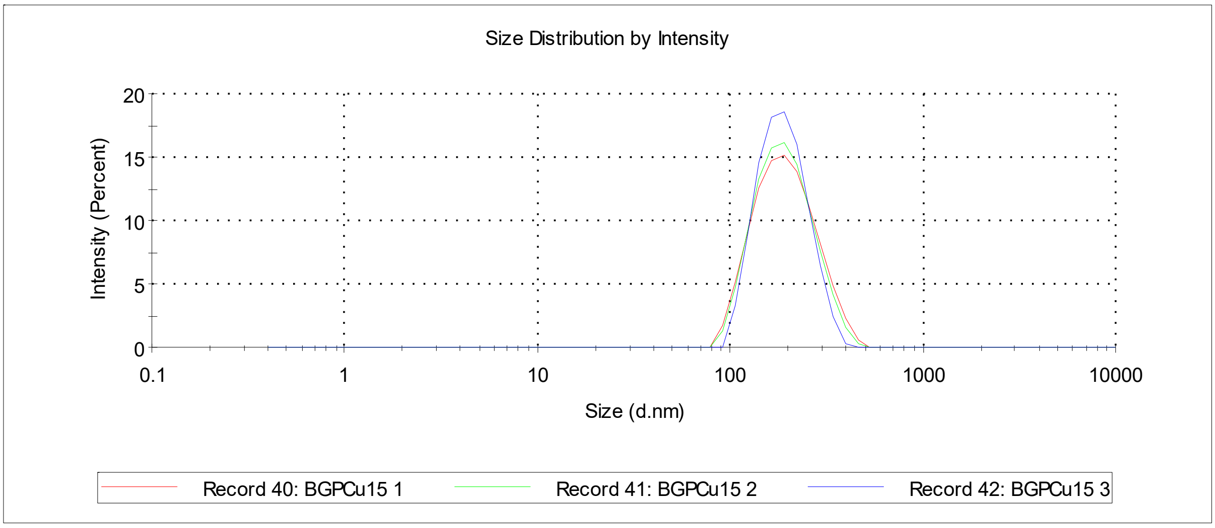
Size Distribution by Intensity
Intensity (Percent)
20
15
10
5
0
0.1
1
10
100
1000
10000
Size (d.nm)
Record 40: BGPCu15 1
Record 41: BGPCu15 2
Record 42: BGPCu15 3

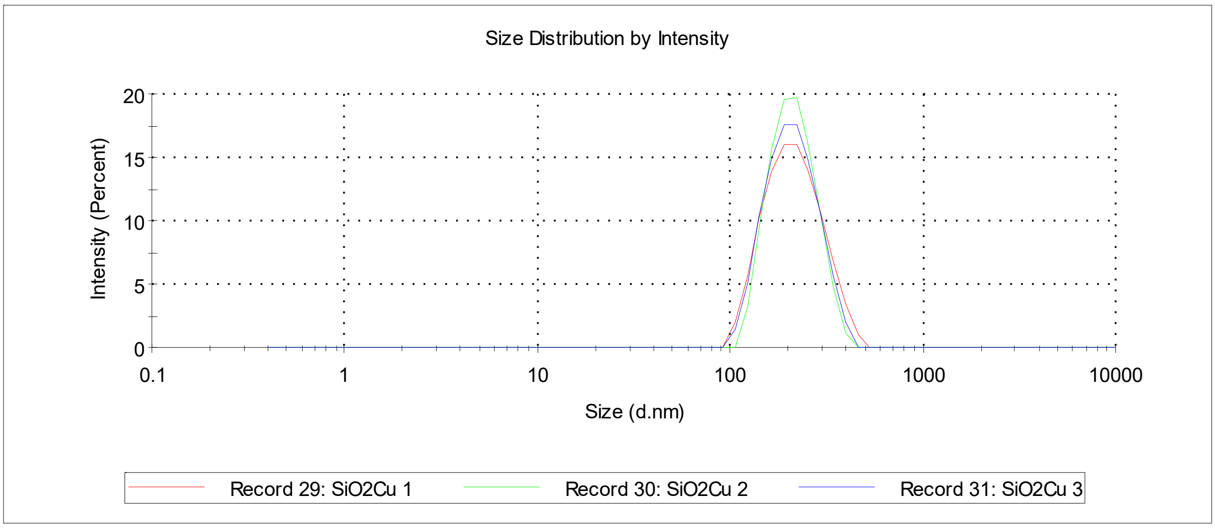
Size Distribution by Intensity
Intensity (Percent)
20
15
10
5
0
0.1
1
10
100
1000
10000
Size (d.nm)
Record 29: SiO2Cu 1
Record 30: SiO2Cu 2
Record 31: SiO2Cu 3

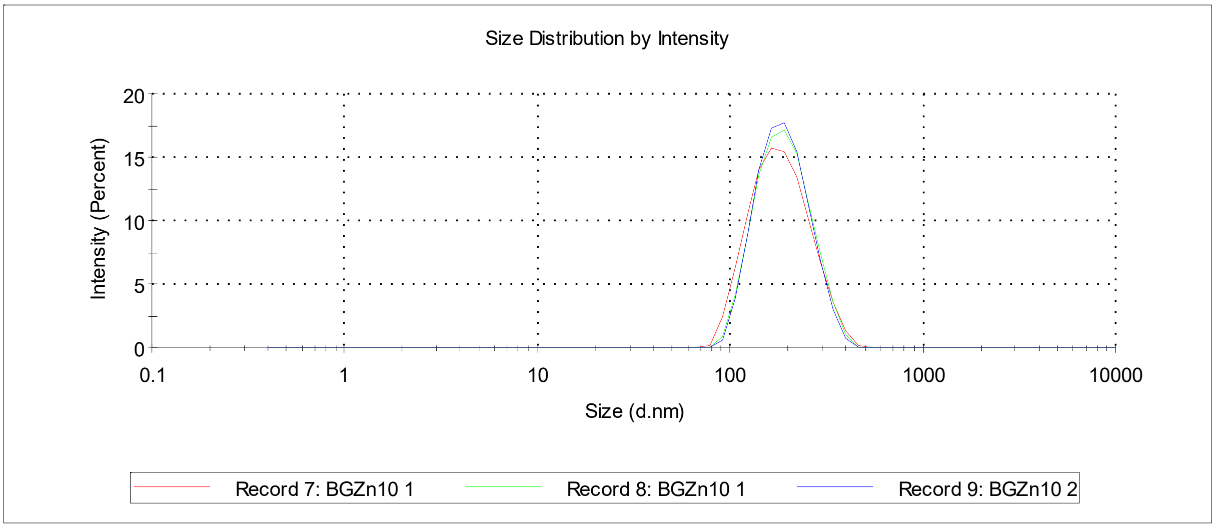
Size Distribution by Intensity
Intensity (Percent)
20
15
10
5
0
0.1
1
10
100
1000
10000
Size (d.nm)
Record 7: BGZn10 1
Record 8: BGZn10 1
Record 9: BGZn10 2

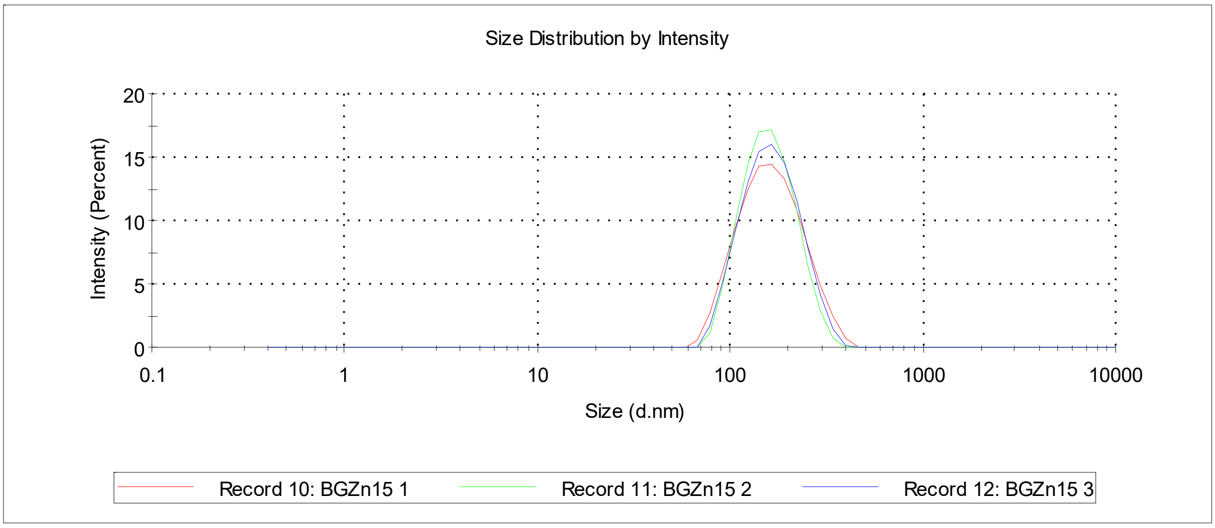
Size Distribution by Intensity
Intensity (Percent)
20
15
10
5
0
0.1
1
10
100
1000
10000
Size (d.nm)
Record 10: BGZn15 1
Record 11: BGZn15 2
Record 12: BGZn15 3

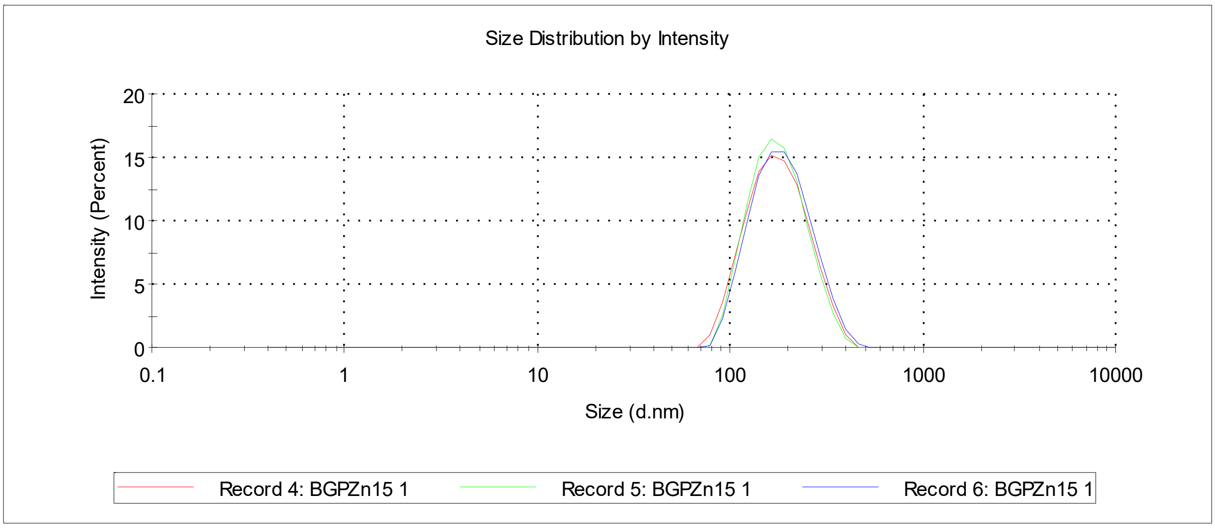
Size Distribution by Intensity
Intensity (Percent)
20
15
10
5
0
0.1
1
10
100
1000
10000
Size (d.nm)
Record 4: BGPZn15 1
Record 5: BGPZn15 1
Record 6: BGPZn15 1

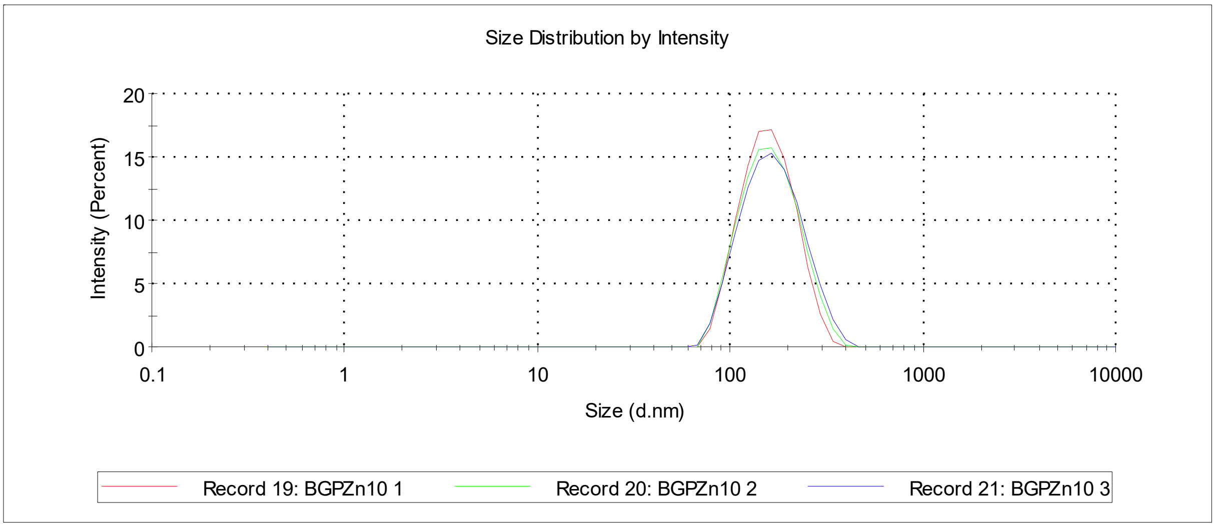
Size Distribution by Intensity
Intensity (Percent)
20
15
10
5
0
0.1
1
10
100
1000
10000
Size (d.nm)
Record 19: BGPZn10 1
Record 20: BGPZn10 2
Record 21: BGPZn10 3
